# Insect herbivory reshapes rhizosphere bacterial and fungal networks in a stage-specific manner

**DOI:** 10.1128/aem.00071-26

**Published:** 2026-04-22

**Authors:** Márcia Leite-Mondin, Priscila A. Auler, Rafael L. Oliveira, Felipe M. R. Barros, Fernando D. Andreote, Marcio C. Silva-Filho

**Affiliations:** 1 Departamento de Genética, Escola Superior de Agricultura Luiz de Queiroz, Universidade de São Paulo 124593 , Piracicaba, Brazil; 2 Departamento de Ciência do Solo, Escola Superior de Agricultura Luiz de Queiroz, Universidade de São Paulo 54538 , Piracicaba, Brazil; The University of Arizona, Tucson, Arizona, USA

**Keywords:** alpha diversity, *Arabidopsis thaliana*, phenology, *Spodoptera frugiperda*

## Abstract

Plants interact with diverse above- and belowground biota, with defense phenotypes shaped by these multiorganismal networks, particularly in the rhizosphere. We tested how insect herbivory reorganizes rhizosphere microbiomes across plant phenology and how it differs from mechanical damage. In *Arabidopsis thaliana* exposed to feeding by the generalist caterpillar *Spodoptera frugiperda* at pre-flowering, flowering, and post-flowering stages, we profiled rhizosphere communities and compared them to mechanically wounded plants. Bacterial alpha diversity was driven largely by phenology, whereas fungal alpha diversity was more sensitive to injury type. PCA revealed clear, treatment-specific separations within each stage, indicating compositional remodeling beyond diversity shifts. Herbivory increased *Streptomyces*, *Sphingomonas*, and *Acinetobacter*, while mechanical injury produced distinct, stage-dependent bacterial signatures, including *Dyella* and *Pseudolabrys*. Fungal responses were stage contingent: fermentative yeasts before flowering and saprotrophic or opportunistic genera after flowering. Co-occurrence networks under both injuries exhibited more positive correlations, with herbivory enhancing bacteria–fungi connectivity. Functional inference showed strong, stage-dependent restructuring: symbiotroph and endophyte guilds clustered at flowering, and post-flowering communities were enriched for hydrogenotrophy, nitrogen fixation, and pathogen/pathotroph functions. Herbivory further elevated inferred chemotrophy, fermentation, and nitrate reduction, whereas mechanical injury reduced chemotrophy, saprotroph abundance, and nitrate-reducing functions. Together, these results indicate that insect herbivory is a biologically distinct disturbance that reorganizes rhizosphere communities and interaction networks in a phenology-dependent manner, underscoring aboveground–belowground linkages with implications for crop resistance and productivity.

**IMPORTANCE:**

Plants rely on soil-dwelling microbes around their roots to grow and defend themselves. Yet we know little about how insects reshape root‑zone communities as plants develop, or whether this differs from simple wounding. We studied *Arabidopsis thaliana* fed on by the generalist caterpillar *Spodoptera frugiperda* before, during, and after flowering and compared these plants to those with mechanical damage. We found that insect feeding, not just injury, acts as a distinct disturbance that reorganizes the root‑zone microbial community in stage‑specific ways. It altered which microbes were present and how they interacted, strengthening links between bacteria and fungi and shifting likely nutrient and defense functions. These results reveal strong connections between aboveground attack and belowground life, with practical implications for breeding and managing crops for resilience and productivity.

## INTRODUCTION

Plants exist within diverse communities of micro- and macroorganisms both above- and belowground (1). To cope with insect herbivory, plants have evolved specialized recognition and defense mechanisms that allow them to detect insect attack and mount responses that differ from those triggered by mechanical damage alone (2, 3). Salivary elicitors from chewing insects play a central role in this discrimination, enabling plants to distinguish true herbivory from physical injury (4). These herbivore-induced defenses not only shape plant resistance but also have triggering effects on the broader community of organisms associated with the plant.

Plant defense is not solely an intrinsic trait of the plant but emerges from interactions with associated organisms. In response to herbivorous insect damage, plants can induce indirect defenses by recruiting the natural enemies of herbivores, such as carnivores, thereby reshaping aboveground insect communities (5). Belowground communities are similarly involved: plants can recruit beneficial soil microorganisms, particularly within the rhizosphere, that enhance resistance to pathogens and herbivores, resulting in the restructuring of belowground microbial communities (1, 6). Recent studies further demonstrate that aboveground herbivore attack can trigger the recruitment of specific rhizobacteria that bolster plant defenses, enhance crop resilience to herbivores, and ultimately improve agricultural productivity (7, 8).

Plant primary and secondary metabolism can be modulated by associated microorganisms (9). A central question in this field is whether specific keystone taxa, or instead interactions among microbial community members, drive changes in insect metabolism and behavior as well as plant defense. Early studies of rhizosphere-mediated plant defense largely focused on individual bacterial species or strains and their effects on plant and insect performance (10, 11). More recently, this reductionist view has shifted toward a community-level perspective in which the rhizosphere microbiome is recognized as a key determinant of plant defense outcomes (8, 12–14). Beyond well-characterized beneficial and pathogenic fungi and bacteria, the rhizosphere harbors a diverse assemblage of lesser-known microorganisms that can directly influence nutrient uptake and plant developmental processes, as well as indirectly protect plants against pathogens (15, 16).

Despite evidence that root exudation shapes the rhizosphere community (17) and plant performance (18) throughout plant development, little is known about how herbivory reshapes rhizosphere microbial communities across distinct plant developmental stages. To address this knowledge gap, we investigated how the rhizosphere microbiota of *Arabidopsis thaliana* responds to attack by the generalist caterpillar *Spodoptera frugiperda* across distinct stages of plant development. We further examined whether insect feeding induces rhizosphere modifications that are distinct from those caused by mechanical damage alone. Specifically, we tested the hypotheses that (i) herbivory modulates the rhizosphere primarily through the presence and activity of the insect rather than through leaf damage *per se*, and (ii) these rhizosphere responses are contingent on plant developmental stage. By dissecting this tripartite interaction among the host plant, the herbivorous insect, and the rhizosphere microbiome, our study provides new insights into how aboveground biotic stress integrates with belowground microbial dynamics, with potential implications for enhancing crop resistance and productivity.

## RESULTS

### Alpha diversity of bacterial and fungal communities across phenological stages and injury treatments

**Fig 1:**
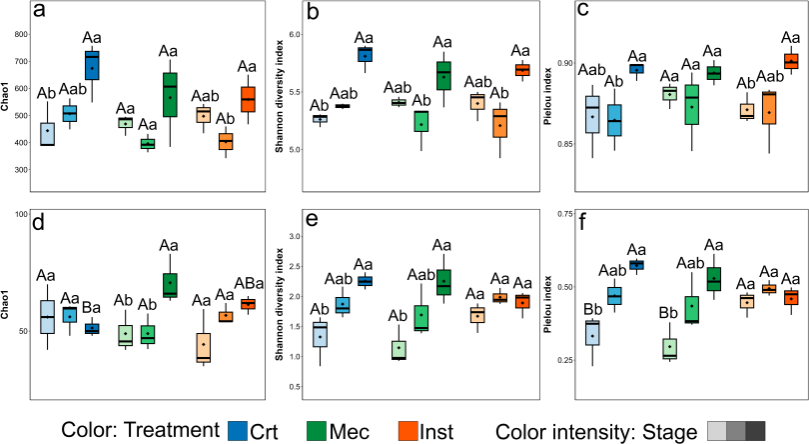
Alpha diversity of bacterial and fungal communities in the *Arabidopsis thaliana* rhizosphere. Alpha diversity indices of bacterial (**a–c**) and fungal (**d–f**) communities are shown for plants subjected to control (Crt), mechanical injury (Mec), or insect herbivory (Inst). Panels display Chao1 richness (**a and d**), Shannon diversity (**b and e**), and Pielou’s evenness (**c and f**). Color intensity within each treatment represents plant phenological stages: pre-flowering (light), flowering (medium), and post-flowering (dark). Different uppercase letters indicate significant differences among phenological stages within the same treatment (main group effect), while different lowercase letters denote significant differences among treatments within the same phenological stage (*P* < 0.05, Kruskal–Wallis test followed by Bonferroni-corrected post hoc comparisons).

After quality filtering, 2,758,401 bacterial 16S rRNA gene reads were retained from 27 samples, averaging 61,287 sequences per sample. These sequences were assigned to 137 bacterial genera distributed across 132 families, 107 orders, 46 classes, and 21 phyla. For fungi, internal transcribed spacer (ITS) sequencing retained 4,974,947 reads after quality filtering, corresponding to an average of 110,554 sequences per sample. The fungal community comprised 203 genera belonging to 166 families, 79 orders, 33 classes, 79 orders, 33 classes, and 13 phyla. To evaluate how injury type influences within-sample microbial diversity across plant development, we analyzed alpha diversity patterns of bacterial and fungal communities within each phenological stage (Fig. 1). This approach allowed us to disentangle the effects of herbivory and mechanical damage while accounting for the strong baseline shifts associated with plant phenology.

**Fig 2:**
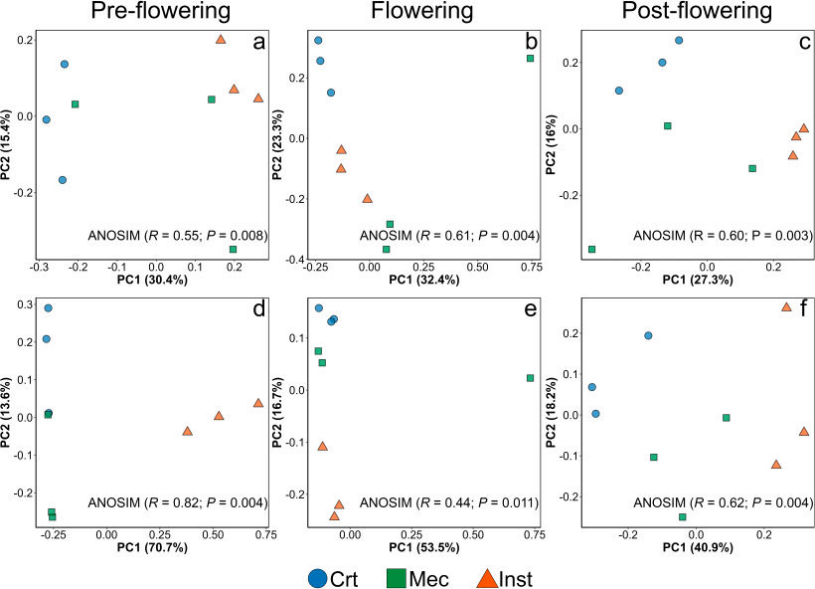
Compositional shifts in bacterial and fungal rhizosphere communities across phenological stages. Principal component analysis of bacterial (**a–c**) and fungal (**d–f**) community composition in the *Arabidopsis thaliana* rhizosphere at (**a and d**) pre-flowering, (**b and e**) flowering, and (**c and f**) post-flowering stages. Treatments include control (Crt), mechanical injury (Mec), and insect herbivory (Inst). Differences in community composition among treatments were evaluated using analysis of similarity (ANOSIM) based on Euclidean distances with 9,999 permutations.

For bacterial communities, Chao1 richness, Shannon diversity, and Pielou’s evenness increased significantly from the pre-flowering to the post-flowering stage in both control (Crt) and insect herbivory (Inst) treatments (*P* < 0.05, Fig. 2a through c). In contrast, under mechanical injury (Mec), Chao1 richness and evenness did not vary across developmental stages (*P* > 0.05), while Shannon diversity was higher at the post-flowering stage than at the flowering stage (*P* < 0.05). No significant differences in bacterial alpha diversity were detected among injury treatments within the same phenological stage (*P* > 0.05).

Fungal communities showed stage-dependent responses to injury type across alpha diversity index (Fig. 2d through f). At the post-flowering stage, Mec plants exhibited higher fungal richness (Chao1) than control plants (*P* < 0.05). In contrast, at the pre-flowering stage, Inst resulted in higher fungal evenness (Pielou’s index) compared with both Crt and Mec plants (*P* < 0.05). Across phenological stages, fungal alpha diversity remained relatively stable under Inst (*P* > 0.05), whereas Mec was associated with increased richness, diversity, and evenness during the post-flowering stage (*P* < 0.05). In Crt plants, Shannon diversity and evenness were also significantly higher at post-flowering than at pre-flowering (*P* < 0.05).

### Compositional shifts in bacterial and fungal rhizosphere communities across phenological stages and injury treatments

To assess overall patterns of rhizosphere community composition, we first conducted a global principal component analysis (PCA) including all samples, followed by two-way PERMANOVA. Across data sets, phenological stage was the primary driver of community structure, while injury treatment had a significant effect only on fungal communities; however, a significant interaction between stage and treatment was detected for both bacteria and fungi (Fig. S1). Given this strong stage-dependent structuring, we then performed PCA analyses within each phenological stage to better resolve treatment-specific effects. Within stages, both bacterial and fungal communities exhibited clear compositional differentiation among injury treatments, indicating that community structure responds to injury type in a stage-dependent manner (Fig. 2; Table S1).

For bacterial communities, analysis of similarity (ANOSIM) revealed significant stage-dependent structuring, with moderate to strong dissimilarity among treatments within each phenological stage (*R* = 0.55–0.61, *P* < 0.01). Pairwise comparisons showed that Inst consistently generated bacterial assemblages that were distinct from Crt communities across all stages (*R* = 1.00; *P* ≤ 0.10). Mec also differed from Crt during the flowering stage (*R* = 0.56, *P* ≤ 0.10), although its effects were generally weaker than those induced by Inst. At the flowering stage, most of the variation along PC1 appeared to be driven by a single Mec sample; although this sample passed all quality control steps and was retained in the analysis, its influence on the observed ordination is noted.

Fungal communities exhibited a strong response to both plant development and injury type. Clear separation among treatments was observed at the pre-flowering and post-flowering stages (*R* = 0.82 and *R* = 0.62, respectively; *P* < 0.01). Pairwise analyses further indicated that Inst generated fungal communities that were highly distinct from Crt at all phenological stages (*R* ≥ 0.95). In addition, during the pre-flowering stage, fungal communities under Mec were also strongly distinct from those under Inst (*R* = 1.00).

### Relative abundance patterns of dominant bacterial and fungal genera across phenological stages and injury treatments

To identify how injury type restructures rhizosphere microbial communities and to pinpoint taxa potentially acting as bioindicators, we analyzed relative abundance patterns of dominant bacterial and fungal genera in linear discriminant analysis effect size (LEfSe) of biomarkers for each treatment at each phenological stage. Across all samples, the dominant bacterial phyla were *Pseudomonadota* (27%), *Actinomycetota* (17%), *Bacillota* (14%), *Bacteroidota* (9%), and *Acidobacteriota* (7%). The fungal community encompassed 13 phyla, 33 classes, 79 orders, 166 families, and 276 genera and was dominated by *Ascomycota* (71%), *Basidiomycota* (25%), and *Mortierellomycota* (3%).

**Fig 3:**
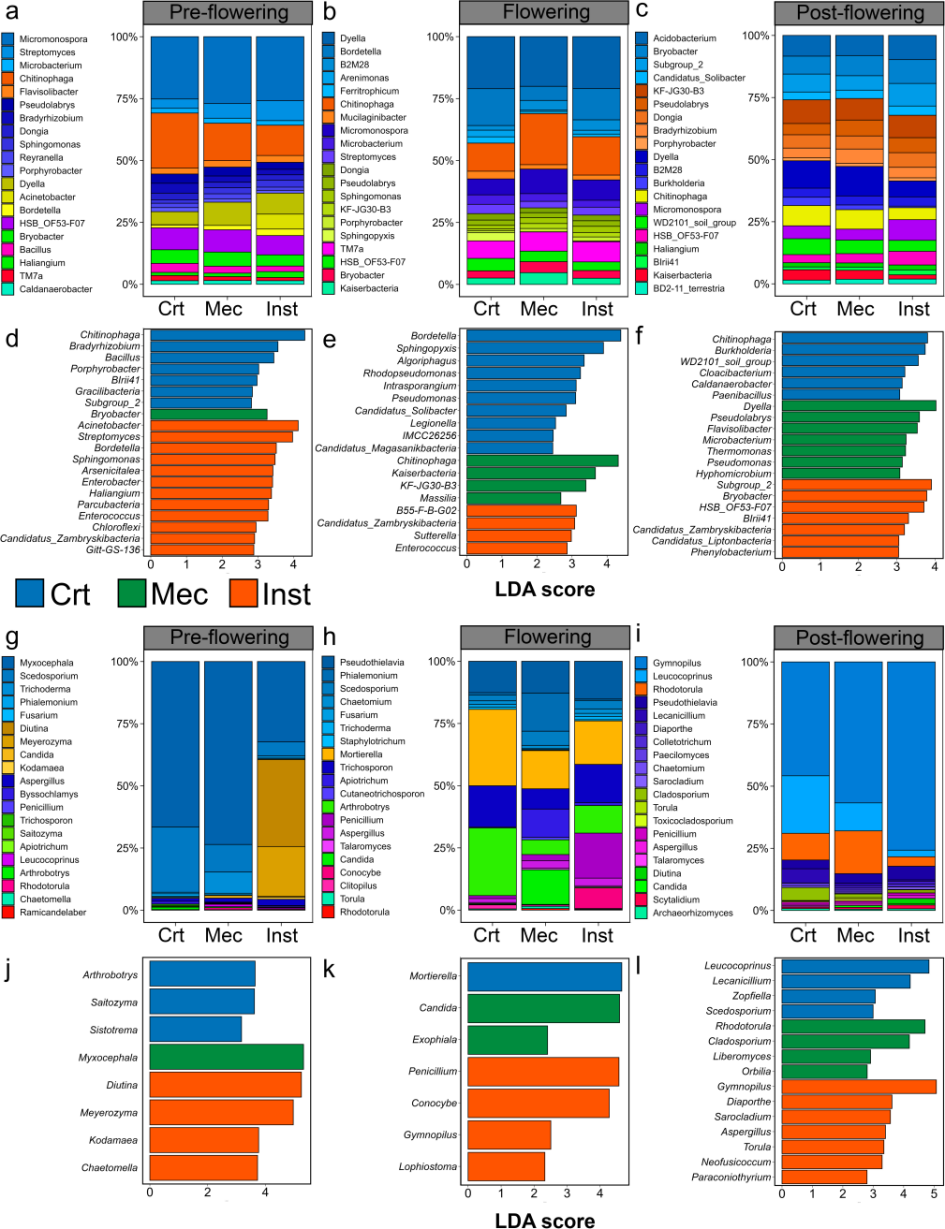
Relative abundance of dominant bacterial and fungal genera across phenological stages. Relative abundances of bacterial (**a–c**) and fungal (**g–i**) genera in the *Arabidopsis thaliana* rhizosphere. Within each stage, treatments include control (Crt), mechanical injury (Mec), and insect herbivory (Inst). Bacterial (**d–f**) and fungal genera (**j–l**) enriched by the treatments as determined by LEfSe analysis (linear discriminant analysis [LDA] score >2 and *P* < 0.05) are shown.

Relative abundance patterns combined with LEfSe analysis revealed consistent and stage-dependent taxonomic restructuring in response to injury treatments, with the strongest divergences typically occurring between Inst and Crt conditions (Fig. 3). Across all developmental stages, bacterial communities were dominated by genera such as *Micromonospora*, *Dyella*, and *Chitinophaga*, although their relative contributions and treatment associations varied with plant phenology. At the pre-flowering stage, both relative abundance profiles and LEfSe identified Inst as a strong driver of bacterial enrichment, particularly for *Acinetobacter*, *Streptomyces*, *Bordetella*, and *Sphingomonas*. The Crt conditions were characterized by higher abundances of *Chitinophaga*, *Bradyrhizobium*, *Bacillus*, and *Porphyrobacter*. The Mec showed a more limited and distinct signature, with only *Bryobacter* enriched, indicating a weaker or more selective filtering effect at this stage.

During the flowering stage, specific shifts were still detectable: *Chitinophaga* was associated with Mec, whereas Crt samples were enriched in taxa such as *Sphingopyxis* and *Bordetella*. In the post-flowering stage, the number of biomarker taxa was similar across treatments, although specific groups differed. Notably, *Dyella* and *Pseudolabrys* were slightly more abundant in Mec. Crt samples were associated with groups such as *Chitinophaga*, *Burkholderia*, and WD2101_soil_group, while Inst showed associations with Subgroup_2, *Bryobacter*, and HSB_OF53-F07.

Despite pronounced differences in the relative abundance of fungi, LEfSe analysis revealed relatively few biomarkers with significant differential abundance. At the pre-flowering stage, Inst samples were characterized by a strong enrichment of fermentative and yeast-associated genera, including *Diutina*, *Meyerozyma*, and *Kodamaea*. In contrast, Crt samples were associated with taxa such as *Arthrobotrys*, *Saitozyma*, and *Symmetrospora*, while Mec samples exclusively showed an increase in *Myxocephala*. During flowering, fungal communities in Mec were distinguished by the biomarkers *Candida* and *Exophiala*, whereas Inst was associated with *Penicillium*, *Conocybe*, and *Gymnopilus*. In Crt samples, only *Mortierella* was enriched. Contrary to the pattern observed for bacteria at the post-flowering stage, Inst exhibited a higher number of biomarkers compared to Mec and Crt. At this stage, Inst consistently enriched saprotrophic and opportunistic genera, including *Gymnopilus*, *Aspergillus*, *Torula*, and *Sarocladium*. In contrast, Crt samples were characterized by taxa such as *Leucocoprinus* and *Leccinellum*, while Mec was associated with *Rhodotorula* and *Cladosporium*.

### Co-occurrence network structure of bacterial and fungal communities under mechanical injury and herbivory

**Fig 4:**
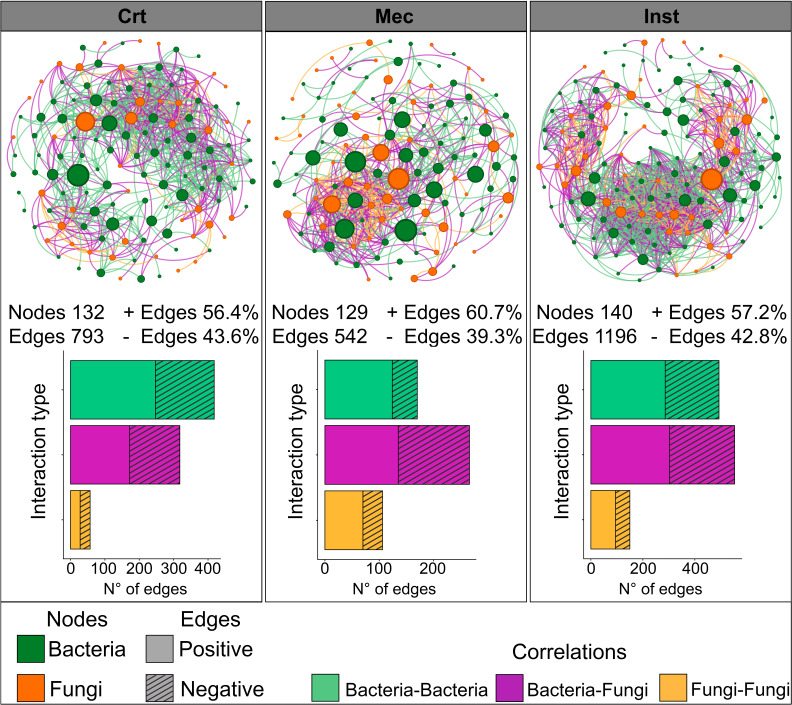
Co-occurrence networks of bacterial and fungal amplicon sequence variants (ASVs) in the *Arabidopsis thaliana* rhizosphere under control (Crt), mechanical injury (Mec), and insect herbivory (Inst) treatments. Networks were constructed by pooling data across all phenological stages, producing one network per treatment. Nodes represent individual ASVs, with size proportional to betweenness centrality and color indicating domain: green for bacteria or orange for fungi. Edges represent robust SparCC correlations (|*r*| > 0.8, *P* < 0.05), with color denoting interaction type: green for bacteria–bacteria, orange for fungi–fungi, or purple for bacteria–fungi.

To explore patterns of microbial co-occurrence in the rhizosphere under different injury treatments, we performed network analyses of bacterial and fungal taxa, which revealed marked structural variation among treatments (Fig. 4). Compared to Crt, Mec reduced network complexity, comprising 129 nodes and 542 edges versus 132 nodes and 793 edges in Crt. In contrast, Inst increased complexity, with 140 nodes and 1,196 edges. Despite these differences, both injury treatments exhibited a higher proportion of positive correlations than Crt (Mec: 60.7%, Inst: 57.2%, and Crt: 56.4%). Cross-domain interactions (bacteria–fungi) were also enhanced under injury. In Crt networks, bacteria–bacteria associations dominated (418 edges, 52.7%), with bacteria–fungi representing 318 edges (40.1%). Mec and Inst networks showed increased cross-domain connectivity, with bacteria–fungi associations accounting for 266 (49.1%) and 553 (46.2%) edges, respectively.

Injury treatments further influenced the identity of keystone genera, defined by highest betweenness centrality (Tables S2 and S3). In Crt and Mec networks, keystone taxa were predominantly bacterial (Crt: eight bacteria, two fungi; Mec: seven bacteria, three fungi), whereas in Inst networks, there was an even distribution between bacteria and fungi (five each). Only two keystone genera, *Thermomonospora* and *Micromonospora*, were shared between Crt and Mec networks.

### Functional analyses of rhizosphere bacterial and fungal communities across plant developmental stages under different foliar injuries

**Fig 5:**
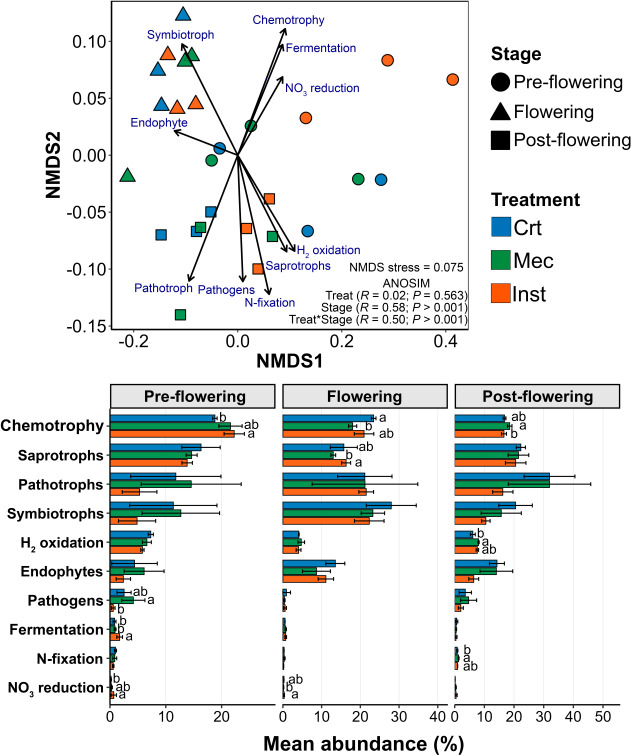
Functional profiles of bacterial and fungal communities in the *Arabidopsis thaliana* rhizosphere across phenological stages and injury treatments. Non-metric multidimensional scaling (NMDS) of bacterial functional groups (FAPROTAX) and fungal guilds (FUNGuild) is shown in the upper panel, based on Bray–Curtis dissimilarities. Vectors indicate the 10 functional categories most strongly correlated with the ordination axes. Lower panels show mean relative abundances (±SE) of dominant functional groups across pre-flowering, flowering, and post-flowering stages for control (Crt), mechanical injury (Mec), and herbivory (Inst) treatments. Different lowercase letters indicate significant differences among treatments within each phenological stage (*P* < 0.05, Kruskal–Wallis test with Bonferroni-corrected post hoc comparisons).

Functional classification of bacterial taxa using FAPROTAX and fungal taxa using FUNGuild revealed additional effects of foliar injury on rhizosphere microbial communities (Fig. 5). FAPROTAX assigned bacterial taxa to 32 functional groups, whereas FUNGuild identified 24 fungal guilds. Non-metric multidimensional scaling (NMDS) based on Bray–Curtis dissimilarities showed an excellent fit (stress = 0.075), indicating clear separation of communities across phenological stages. The ordination highlighted the functional groups and guilds contributing most to community variation. ANOSIM revealed no significant effect of injury treatment alone (*R* = 0.02, *P* = 0.563), whereas phenological stage (*R* = 0.58, *P* < 0.001) and the stage × treatment interaction (*R* = 0.50, *P* < 0.001) were highly significant. Communities at the flowering stage clustered with symbiosis and plant association-related functions, including symbiotroph and endophyte guilds. In contrast, post-flowering communities formed a distinct cluster associated with H_2_ oxidation, saprotroph abundance, N fixation, pathogen, and pathotroph functions.

Examination of mean relative abundances revealed treatment-specific functional responses. At the pre-flowering stage, Inst increased chemotrophy, fermentation, and NO_3_ reduction, and during the flowering stage, it enriched saprotroph abundance, and NO_3_ reduction functions. Mec reduced chemotrophy, saprotroph abundance, and NO_3_ reduction during flowering but enhanced chemotrophy, H_2_ oxidation, and N fixation at the post-flowering stage.

## DISCUSSION

### Phenological stage and injury type differentially modulate rhizosphere bacterial and fungal diversity and community structure

Plant development is accompanied by shifts in carbon allocation, nutrient demand, and defense investment, collectively shaping rhizosphere conditions and microbial assembly over the plant life cycle (19). Accordingly, plant phenological stage is a major driver of rhizosphere microbial diversity and community structure, largely mediated through changes in root exudation and plant–microbe interactions (20). Our results support this framework but further demonstrate that bacteria and fungi respond asymmetrically to phenology and plant injury, reflecting fundamental differences in their ecological strategies and host dependence.

Bacterial alpha diversity was primarily structured by plant phenological stage, with richness and diversity consistently higher at post-flowering compared to pre-flowering in both control and herbivory treatments (Fig. 1a through c). This pattern aligns with previous studies reporting stage-dependent bacterial community assembly in crop systems (21–23). The increase in bacterial alpha diversity at the post-flowering stage likely reflects shifts in root-derived resources. As plants mature and senesce, they modify exudation profiles, releasing distinct blends of compounds and specific phytochemicals that orchestrate rhizosphere microbiome assembly (17). This enhanced substrate heterogeneity, including secondary metabolites and compounds from fine root turnover, creates a wider array of ecological niches (13), supporting a more diverse bacterial community.

In contrast, under mechanical injury, bacterial richness and evenness remained comparatively stable across phenological stages. This pattern indicates that non-specific wounding constrained the expression of phenology-driven alpha diversity shifts without suppressing community reassembly. Although mechanical damage activates defense pathways partially overlapping with herbivory-induced responses, it often fails to elicit the full suite of plant responses triggered by insect attack (24, 25). Consequently, mechanical injury may reduce variation in root-derived resources across developmental stages, limiting niche differentiation detectable at the level of richness and evenness. Importantly, stabilization of alpha diversity does not imply compositional uniformity: bacterial community structure under mechanical injury remained distinct from controls across all stages, indicating substantial taxonomic turnover within a constrained diversity envelope.

Fungal alpha diversity, by contrast, was more strongly regulated by injury type than phenological stage. Herbivory maintained relatively stable fungal diversity across stages, whereas mechanical injury promoted higher richness, Shannon diversity, and evenness at post-flowering (Fig. 1d through f). These results suggest that fungi are particularly sensitive to the nature of plant damage, consistent with reports showing strong fungal responses to herbivore- and injury-induced host changes (26). The pronounced fungal response to treatment indicates a tighter coupling between fungal community dynamics and host-derived signals compared to bacteria.

PCA revealed clear separation of bacterial and fungal communities among treatments within each phenological stage (*P* < 0.01, Fig. 2), indicating that plant injury reshaped community composition beyond shifts in diversity metrics alone. For bacteria, herbivory consistently generated communities distinct from controls across all stages, whereas mechanical injury produced intermediate profiles. This suggests that part of the bacterial community responds to injury *per se*, while another fraction responds specifically to insect-associated cues.

Fungal community structure exhibited even stronger treatment-driven differentiation, particularly at pre- and post-flowering stages (Fig. 2d through f). Herbivory redefined fungal community composition relative to controls, regardless of phenological stage, reinforcing the view that fungi are highly sensitive to host alterations and the biochemical nature of plant damage. Such structural shifts likely reflect plant-mediated feedback that prioritizes defense over growth following insect attack, involving herbivore-induced modifications of root exudate chemistry to recruit beneficial fungi and shape the rhizosphere microbiome (27). Insect herbivory induces systemic signaling that redirects resources toward defense compound synthesis, alters root development, and modifies root exudation profiles (28–30). Additionally, insect oral secretions and localized tissue disruption introduce biochemical cues that further shape belowground signaling and microbial recruitment (31, 32).

Also, it should be noted that these findings were obtained in *A. thaliana*, a short-lived species. In longer-lived species sampled at comparable chronological time points, where plants may undergo relatively little physiological change over those intervals, it remains unclear whether microbial communities would still exhibit temporal shifts as they assemble.

### Relative abundance patterns of dominant bacterial and fungal genera across phenological stages and injury treatments

Clear shifts were observed in the dominant bacterial and fungal genera across developmental stages and treatments, with the most pronounced differences occurring under herbivory. Among bacterial genera, *Streptomyces*, *Sphingomonas*, and *Acinetobacter* increased in abundance under herbivory during the pre-flowering stage, whereas *Chitinophaga*, *Bacillus*, and *Porphyrobacter* decreased relative to control.

The genera enriched under herbivory are well-documented responders to insect or pathogen attack. *Streptomyces* spp. correlate with resistance to pathogen invasion and can trigger salicylic acid signaling and induced systemic resistance, activating plant defense metabolism (33–35). *Sphingomonas* abundance has been associated with herbivory-induced microbiome shifts, enhancing plant growth, resistance to biotic stress, and participation in nitrogen cycling through fixation, nitrification, and denitrification (36, 37). Herbivory can increase rhizosphere nitrogen through nitrogen-rich frass deposition, which originates from incompletely assimilated plant compounds, thus stimulating microbial activity and accelerating nitrogen cycling (38, 39). *Acinetobacter* may act directly on insects, facilitating adaptation to plant defenses, as shown in *Camellia* weevils acquiring gut microbiota from soil-derived *Acinetobacter*, enabling saponin degradation and persistence on chemically defended fruits (40). This supports the idea of host manipulation by insects and aligns with our observation of higher *Acinetobacter* abundance in herbivory-treated plants.

Mechanical injury produced genus-specific, phase-dependent signatures distinct from those induced by herbivory. *Dyella* and *Pseudolabrys* increased under mechanical injury relative to both herbivory and controls during the post-flowering stage. *Dyella* exhibits metabolic versatility relevant to plant interactions, biodegradation, and nutrient recycling, with some species promoting lateral root elongation and seedling biomass (41). *Pseudolabrys* has been linked to soil organic matter and plant metabolic processes involving hormones, alkaloids, nitrogen, and aroma precursors such as chlorophyll (42, 43), potentially modulating plant responses to mechanical foliar damage. Furthermore, the amount of leaf consumed by the insect was not identical to that removed mechanically, so these differences, even if subtle, can influence the bacterial community.

Fungal responses were strongly influenced by herbivory. During pre-flowering, herbivory increased the abundance of fermentative yeasts, including *Diutina*, *Meyerozyma*, and *Kodamaea* (*Ascomycota*). This enrichment may relate to elevated nitrogen availability from insect frass, which can stimulate *Ascomycota* proliferation and enhance root nutrient absorption, nitrogen utilization, and substrate provision for organic synthesis (44, 45). Herbivory also reduced the abundance of *Arthrobotrys*, a fungal genus involved in nematode control and pathogen defense (46, 47), reflecting the specificity of herbivory-induced signaling pathways compared with pathogen attack (48).

After flowering, herbivory markedly increased saprotrophic and opportunistic fungi, including *Gymnopilus*, *Aspergillus*, *Torula*, and *Sarocladium*. This enrichment likely reflects combined effects of plant senescence, root exudates rich in amino acids, phenolics, organic acids, and herbivory-induced stimulation of exudation and tissue breakdown (17). Accordingly, these taxa were most abundant in rhizospheres of insect-attacked plants during late developmental stages.

### Insect herbivory promotes bacterial–fungal correlations in soil microbial networks

Co-occurrence network analyses capture not only “who is there” but also “who potentially interacts with whom” in the rhizosphere. This is particularly important because the emergent functions of the root microbiome arise from interactions among bacteria and fungi, not merely from taxonomic composition. By examining these putative interactions under different types of plant injury, we gain a more nuanced understanding of how microbial relationships respond to aboveground damage.

Herbivory yielded the greatest increase in network complexity, with more nodes and, notably, more edges, indicating tighter coordination among taxa. This enhanced complexity is often associated with greater network stability and functional intricacy (49). Both injury treatments also increased the proportion of positive correlations relative to controls, suggesting that stress or lesion conditions promote more aligned co-variation among taxa. In rhizosphere microbiomes, moderate stress has been reported to elevate positive associations in co-occurrence networks (50, 51), consistent with our observations.

Notably, herbivory enhanced cross-domain connectivity between bacteria and fungi, reducing the dominance of bacteria-only interactions seen in controls. This stronger cross-domain coupling may reflect increased functional interactions, whereby bacteria and fungi exploit or modulate the same plant-derived resources or response pathways (52, 53).

### Functional analyses of rhizosphere bacterial and fungal communities reveal stage-dependent microbiome shifts and injury-driven functional modulation

NMDS analyses across plant developmental stages revealed distinct functional clustering in the rhizosphere microbiome. During flowering, plant–microbe association guilds such as symbiotrophs and endophytes clustered together, reflecting the reconfiguration of plant resource allocation and root exudation to favor beneficial microbial interactions during the reproductive transition (29, 54). In contrast, during the post-flowering stage, a distinct cluster emerged, associated with H_2_ oxidation (hydrogenotrophy), saprotrophs (decomposers), nitrogen fixation, pathogens, and pathotroph guilds. This shift likely reflects senescence-driven inputs of necromass and late-stage exudates, as well as physiological changes that favor decomposition, nitrogen cycling, and increased pathogen pressure (17).

Regarding treatment effects, herbivory increased the inferred representation of chemotrophy, fermentation, and NO_3_^−^ reduction. Herbivore attack can elevate sulfur and nitrogen levels in the rhizosphere through deposition of nitrogen-rich frass (38), which, combined with tissue damage, likely promotes fermentative metabolisms, nitrate-reductive processes, and the expansion of chemotrophic microbial groups (55).

By contrast, mechanical injury reduced chemotrophy, saprotrophs, and NO_3_^−^-associated taxa. Unlike herbivory, mechanical damage does not contribute frass-derived nitrogen nor does it elicit the full spectrum of defense-related exudates that stimulate specific microbial functions (48, 56). However, during post-flowering senescence, even mechanically injured plants exhibited increased chemotrophy, H_2_ oxidation, and nitrogen fixation, likely driven by age-dependent physiological transitions that favor chemotrophy, hydrogenotrophy, and nutrient cycling. Overall, the “functional signature” of the rhizosphere microbiome is strongly stage dependent. While injury type alone does not globally reorganize functional networks, its interaction with plant developmental stage can redirect specific pathways, including decomposition, fermentation, nitrate reduction, hydrogen oxidation, and nitrogen fixation, highlighting the combined influence of plant ontogeny and aboveground damage on rhizosphere functionality.

It is worth highlighting that this study has limitations that point to clear avenues for future studies. Volatile organic compounds (VOCs) may influence rhizosphere reshaping, and although VOCs transfer from attacked to unattacked plants has been reported (4, 57), it was not quantified here. However, microbial volatile organic compounds have been chiefly associated with root-feeding herbivores (58), while our findings concern aboveground herbivory. Furthermore, characterization of root exudation across different developmental stages may advance understanding of how the rhizosphere microbiome assembles and functions under herbivory. Finally, because the microbial diversity in our laboratory substrate differs from that in natural field soils, our findings may not directly translate to field settings, particularly with respect to the specific microbial taxa involved. Nevertheless, this study demonstrates that herbivory, even when it happens to aboveground tissues, can modulate belowground community dynamics in a phenology-dependent manner. These observations generate testable hypotheses about additional plant defense strategies against herbivory that should be validated in future studies under natural field conditions and across multiple plant species.

## MATERIALS AND METHODS

### *Arabidopsis thaliana* COL-0 growth conditions

*Arabidopsis thaliana* ecotype Columbia (Col-0, wild type) seeds were obtained from self-fertilized seed stocks maintained in the seed bank of the Plant Molecular Biology Laboratory, Department of Genetics, ESALQ/USP. The original parental seed stock was purchased from Lehle Seeds (Round Rock, TX, USA). Seeds were surface-sterilized in a 2.5% sodium hypochlorite solution and rinsed three times with sterile water prior to sowing. Seeds were then sown directly into a non-sterile substrate composed of vermiculite (Plantmix-Trimix) and peat moss (Basaplant Florestal) mixed at a 1:1 (vol/vol) ratio. The substrate was prepared as a single homogeneous batch.

**Fig 6:**
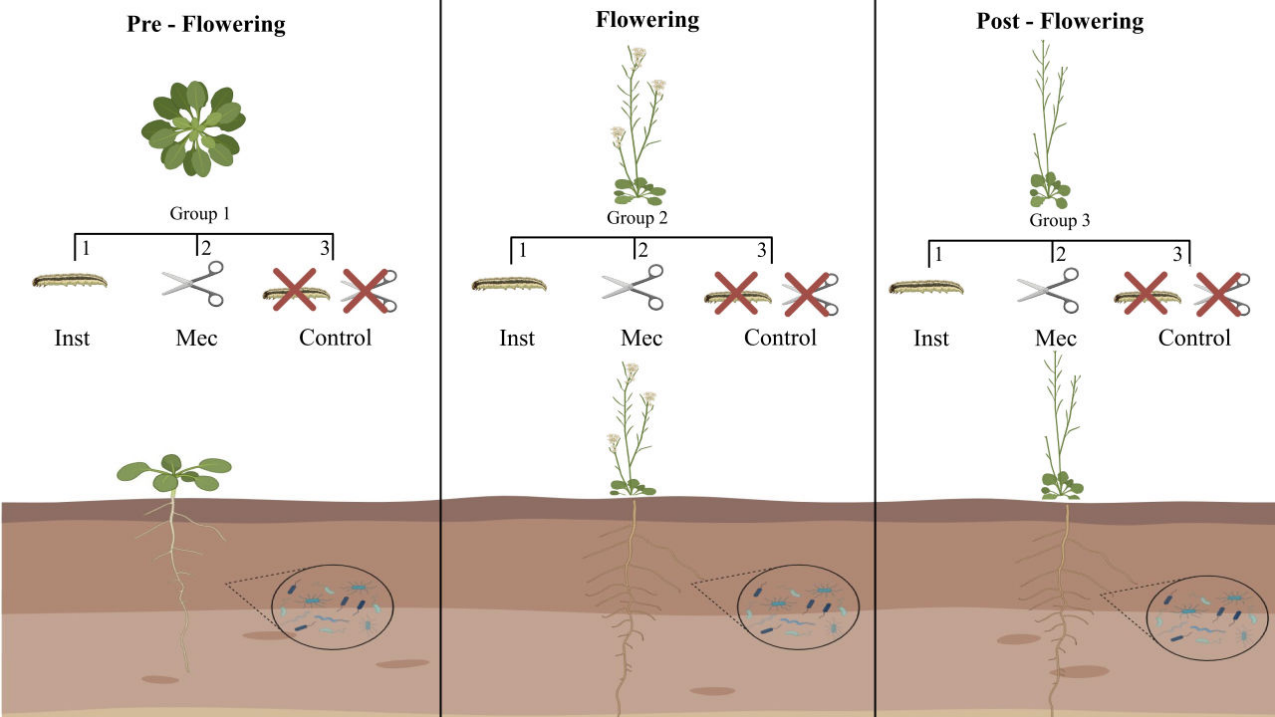
Experimental design. *Arabidopsis thaliana* plants were assigned to three developmental groups: pre-flowering (group 1), flowering (group 2), and post-flowering (group 3). Within each group, plants were further subdivided into three treatment subgroups: insect herbivory (Inst), mechanical wounding (Mec), and control (Crt). Plants in the Inst subgroup were exposed to *Spodoptera frugiperda* herbivory for 48 h, whereas plants in the Mec subgroup received standardized mechanical damage (indicated by scissors). Control plants remained untreated. Rhizosphere samples were collected from all treatments 48 h after the onset of the herbivory treatment.

Plants were categorized into three developmental stages: pre-flowering (15 days after sowing), flowering (25 days after sowing), and post-flowering (35 days after sowing). Within each developmental stage, plants were further subdivided into three treatment groups: insect herbivory (Inst), mechanical damage (Mec), and untreated control (Crt). Plants in the Inst group were exposed to insect herbivory for 48 h, whereas those in the Mec group received standardized mechanical wounding consisting of a single cut along the leaf margin using sterile scissors. Control plants were left untreated, with no wounding or insect exposure (Fig. 6). Each treatment group comprised three biological replicates (*n* = 3).

All plants were grown individually in 400 cm³ pots and maintained under identical controlled environmental conditions in a growth chamber (25°C ± 1°C, 16 h photoperiod, light intensity of approximately 35 µmol m⁻² s⁻¹). The experiment followed a completely randomized design.

### Insect rearing and herbivory treatment

Third-instar *Spodoptera frugiperda* (Lepidoptera: Noctuidae) larvae were used for herbivory assays. Larvae were obtained from the Laboratory of Insect Biology, Department of Entomology and Acarology, Luiz de Queiroz College of Agriculture, University of São Paulo (Piracicaba, SP, Brazil). Insects were reared under controlled conditions at 25°C ± 1°C, 60% ± 10% relative humidity, and a 14:10 h light:dark photoperiod. For herbivory treatments, five larvae were placed directly onto the leaves of each *Arabidopsis thaliana* plant in the insect (Inst) treatment group and allowed to feed for 48 h at each plant developmental stage tested.

### Rhizosphere sample collection

Rhizosphere samples from *Arabidopsis thaliana* were collected 48 h after treatment initiation in all experimental groups (insect herbivory, mechanical damage, and control). For each developmental stage, the experiment was initiated (t₀) when plants reached the corresponding phenological stage: pre-flowering (15 days after sowing), flowering (25 days after sowing), or post-flowering (35 days after sowing). Rhizosphere soil was defined as soil tightly adhering to the root surface. First, the plant was removed along with a 2 mm layer of soil surrounding the plant stem. This removal was performed using a spatula inserted into the soil surface. After sampling, the soil adhering to the root was manually detached, without washing. Samples were transferred to sterile 15 mL tubes, immediately flash-frozen in liquid nitrogen, and stored at −80°C until DNA extraction.

### DNA extraction and amplicon sequencing

Total DNA was extracted from 0.25 g of rhizosphere soil using the DNeasy PowerSoil Kit (QIAGEN, Carlsbad, CA, USA) following the manufacturer’s instructions. The V4 region of the bacterial 16S rRNA gene was amplified using primers 515f (GTGYCAGCMGCCGCGGTAA) and 806r (GGACTACNVGGGTWTCTAAT) (59, 60). Fungal community composition was assessed by amplifying the ITS region of the rRNA using primers ITS1F (CTTGGTCATTTAGAGGAAGTAA) and ITS2 (GCTGCGTTCTTCATCGATGC) (61). A total of 27 bacterial 16S rRNA gene and 27 fungal ITS amplicon samples were sequenced, with five replicates per treatment.

Amplicon libraries were prepared according to the Illumina 16S Metagenomic Sequencing Library Preparation protocol. PCR reactions (20 µL) were performed using KAPA HiFi DNA Polymerase, with 2 µL of template DNA per reaction. Thermal cycling conditions consisted of an initial denaturation at 95°C for 3 min, followed by 25 cycles of 95°C for 20 s, 55°C for 10 s, and 72°C for 20 s, with a final extension at 72°C for 5 min.

PCR products were quantified using Qubit, pooled at equimolar concentrations, and sequenced on an Illumina MiSeq platform using 300 bp paired-end reads.

### Data processing and bioinformatic analyses

Sequencing data were processed using Quantitative Insights Into Microbial Ecology (QIIME 2, version 2024.2 [62]). Paired-end reads were quality-filtered, trimmed, denoised, and dereplicated using the q2-dada2 plugin to infer amplicon sequence variants (ASVs) (63). Taxonomic assignment of bacterial ASVs was performed using the SILVA reference database (version 138 [64]), while fungal ASVs were classified using the UNITE database (version 8 [65]).

Downstream analyses were conducted in R (version 4.5.1). Microbiome data sets were imported into the phyloseq package (66) for data handling and visualization. Alpha diversity indices (Chao1 richness, Shannon diversity, and Pielou’s evenness) were calculated using the microbiome package (67). Statistical differences among treatments were assessed using Kruskal–Wallis tests followed by Dunn’s post hoc tests with Bonferroni correction (68).

Community composition was visualized using PCA based on Hellinger-transformed ASV tables. Differences in community structure among treatments and developmental stages were tested using analysis of similarities (ANOSIM) with 9,999 permutations, implemented in the vegan package (69).

Relative abundance analyses were conducted using the microeco package (70), with ASVs aggregated at the genus level. The 20 most abundant genera per treatment were visualized using the ggnested package. Additionally, LEfSe was performed to identify differentially abundant genera among treatments, considering only taxa with linear discriminant analysis scores above 2 and *P* < 0.05.

Co-occurrence network analyses were performed using the trans_network class in microeco, retaining ASVs with a minimum relative abundance of 0.05%. Correlations among taxa were inferred using the SparCC method implemented in the SpiecEasi package (71). Robust associations were retained using a correlation threshold of |*r*| ≥ 0.8 and a significance cutoff of *P* < 0.05. Network modules were identified using the Louvain clustering algorithm. Network visualization and topological metrics, including number of nodes and edges, average degree, modularity, and community structure, were generated using Gephi (72).

Functional annotation of bacterial communities was performed using FAPROTAX (73), while fungal ecological guilds were assigned using FUNGuild (74), both implemented via the microeco package. Functional categories were aggregated into seven bacterial groups (chemotrophy, hydrogen oxidation, ureolysis, nitrate reduction, aromatic compound degradation, nitrogen fixation, and sulfide oxidation) and six fungal guilds (saprotrophs, pathogens, endophytes, mycorrhizal fungi, pathotrophs, and symbiotrophs). Relative abundances were calculated as abundance-weighted means.

Bacterial and fungal functional matrices were merged, Hellinger-transformed, and ordinated using NMDS based on Bray–Curtis dissimilarity. Functional groups significantly correlated with ordination space (envfit, *P* < 0.05) were identified, and the 10 strongest correlations were visualized. Differences among treatments, plant developmental stages, and their interaction were tested using ANOSIM with 9,999 permutations. Differences in functional group abundances were assessed using Kruskal–Wallis tests (*P* < 0.05), followed by Dunn’s post hoc tests with Bonferroni correction.

### Quantitative PCR analysis

Absolute abundances of bacterial and fungal communities were quantified by real-time quantitative PCR targeting the bacterial 16S rRNA gene and the fungal ITS region. Reactions were performed on a StepOne Real-Time PCR System (Applied Biosystems) using Maxima SYBR Green/ROX Master Mix in a final reaction volume of 25 µL, with all samples analyzed in technical triplicate. Gene copy numbers were expressed as copies g⁻¹ of soil. Bacterial 16S rRNA genes were amplified using primers P2 Eub518R (ATTACCGCGGCTGCTGG) and P1 341F (CCTACGGGNGGCWGCAG) (75), while fungal ITS regions were amplified using primers ITS1 (TCCGTAGGTGAACCTGCGG) and 5.8S (CGCTGCGTTCTTCATCGA) (76). Amplification conditions for bacterial 16S rRNA consisted of an initial denaturation at 95°C for 3 min, followed by 35 cycles of 94°C, 55°C, and 72°C for 30 s each. Fungal ITS amplification included an initial incubation at 50°C for 2 min and 95°C for 2 min, followed by 44 cycles of 95°C for 15 s and 60°C for 1 min.

Amplification specificity was confirmed by melt-curve analysis. Standard curves were generated from 10-fold serial dilutions (10⁻² to 10⁻⁸) of purified PCR products, yielding coefficients of determination (*R*²) greater than 0.98. Absolute gene copy numbers were calculated using the StepOne software.

Differences among experimental groups were assessed by one-way analysis of variance, followed by Tukey’s post hoc test (*P* < 0.05), using PAST software. Bacterial 16S rRNA and fungal ITS gene abundances are presented in Fig. S2 and S3.

## Data Availability

The original raw data (sequencing reads) are available in the NCBI database under BioProject number PRJNA1442228.

## SUPPLEMENTAL MATERIAL

Supplemental material aem.00071-26-s0001.pdf Tables S1 to S3; Fig. S1 to S3.

ASM does not own the copyrights to Supplemental Material that may be linked to, or accessed through, an article. The authors have granted ASM a non-exclusive, world-wide license to publish the Supplemental Material files. Please contact the corresponding author directly for reuse.
