# Supplementary material for: Insect herbivory reshapes rhizosphere bacterial and fungal networks in a stage-specific manner
Source: Appl Environ Microbiol. 2026 Apr 22;92(5):e00071-26. doi: 10.1128/aem.00071-26 (PMC13188856; doi:10.1128/aem.00071-26)
Supplement: Supplemental material — Tables S1 to S3; Fig. S1 to S3. [file aem.00071-26-s0001.pdf]

Table S1: Analysis of similarities (ANOSIM) results for bacterial and fungal community structure across treatments (control, mechanical injury, and herbivory) at different plant developmental stages (pre-flowering, flowering, and post-flowering).

Bacteria			
Pre-flowering			
Group1	Group2	R	P_adj
Control	Mechanical injury	0.259	0.3
Control	Herbivory	1.000	0.1
Mechanival injury	Herbivory	0.333	0.1
Flowering			
Control	Mechanical injury	0.556	0.1
Control	Herbivory	1.000	0.1
Mechanival injury	Herbivory	0.444	0.1
Post-flowering			
Control	Mechanical injury	0.370	0.1
Control	Herbivory	1.000	0.1
Mechanival injury	Herbivory	0.370	0.1

Fungi			
Pre-flowering			
Group1	Group2	R	P_adj
Control	Mechanical injury	0.407	0.2
Control	Herbivory	1.000	0.1
Mechanival injury	Herbivory	1.000	0.1
Flowering			
Control	Mechanical injury	0.259	0.2
Control	Herbivory	0.953	0.1
Mechanival injury	Herbivory	0.185	0.2
Post-flowering			
Control	Mechanical injury	0.481	0.1
Control	Herbivory	0.963	0.1
Mechanival injury	Herbivory	0.407	0.1

Table S2: The topological properties of microbial community networks.

Network properties	Independent of the phenological stage		
	**Control**	**Mechanical injury**	**Herbivory**
Number of nodes [a]	132	129	140
Number of edges [b]	793	542	1196
Positive edges [c]	447	329	684
Negative edges [d]	346	213	512
Modularity [e]	0.479	0.456	0.435
Number of communities [f]	8	9	9
Network diameter [g]	8	9	8
Average path length [h]	3.231	3.546	2.854
Average degree [i]	12.015	8.403	17.086
Average clustering coefficient [j]	0.603	0.570	0.629

[a] Microbial community (at genus level) with at least one significant ($p < 0.05$) and strong (SparCC > 0.8 or < -0.8) correlation.

[b] Number of connections/correlations obtained by SparCC analysis.

[c] SparCC positive correlation (> 0.8 with $p < 0.05$).

[d] SparCC negative correlation (< -0.8 with $p < 0.05$).

[e] The capability of the nodes to form highly connected communities, that is, a structure with high density of between nodes connections (inferred by Gephi).

[f] A community is defined as a group of nodes densely connected internally (Gephi).

[g] The longest distance between nodes in the network, measured in number of edges (Gephi).

[h] Average network distance between all pair of nodes or the average length off all edges in the network (Gephi).

[i] The average number of connections per node in the network, that is, the node connectivity (Gephi).

[j] How nodes are embedded in their neighborhood and the degree to which they tend to cluster together (Gephi).

Table S3: Top ten genera with the highest betweenness centrality values in the microbial co-occurrence network.

Independent of the phenological stage			
**Treatment**	**Genus**	**Betweenness Centrality**	**Degree**
**Control**	Bac_Candidatus Kaiserbacteria	1145	20
	Fun_Scedosporium	968	22
	Bac_Thermomonospora	738	29
	Bac_Bacillus	569	11
	Fun_Lecanicillium	527	29
	Bac_Edaphobacter	510	22
	Bac_Dongia	496	26
	Bac_Micromonospora	458	17
	Bac_Candidatus Saccharimonas	416	6
	Bac_Bauldia	379	6
**Mechanical injury**	Bac_WD2101	785	9
	Fun_Trichosporon	757	26
	Bac_SM1A02	733	21
	Bac_Acidobacterium	668	28
	Fun_Gymnopilus	591	29
	Fun_Myxocephala	551	13
	Bac_Micromonospora	543	13
	Bac_Fimbriimonas	541	6
	Bac_IMCC26256	532	4
	Bac_Thermomonospora	488	33
**Herbivory**	Fun_Trichosporon	954	38
	Bac_Chloroflexi	588	31
	Bac_Blrii41	544	34
	Bac_Microbacterium	446	49
	Bac_Flavisolibacter	408	30
	Bac_Dyella	400	14
	Fun_Gymnopilus	395	47
	Fun_Myxocephala	374	25
	Fun_Mortierella	370	24
	Fun_Penicillium	349	23

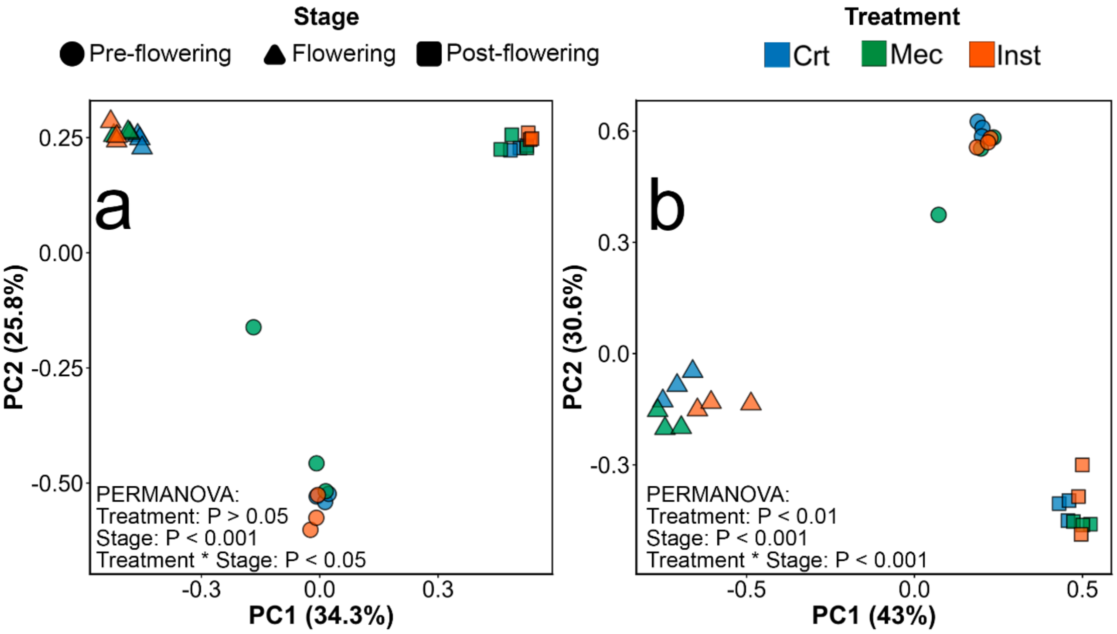

Fig. S1: Global patterns of bacterial and fungal community composition across phenological stages and injury treatments. Principal component analysis (PCA) of bacterial (a) and fungal (b) rhizosphere community composition in *Arabidopsis thaliana*, including all samples across phenological stages and treatments. Symbols represent phenological stages (pre-flowering, flowering, and post-flowering), and colors indicate treatments: control (Crt), mechanical injury (Mec), and insect herbivory (Inst). Differences in community composition were evaluated using two-way PERMANOVA based on Hellinger-transformed data with 9,999 permutations, testing the effects of phenological stage, injury treatment, and their interaction.

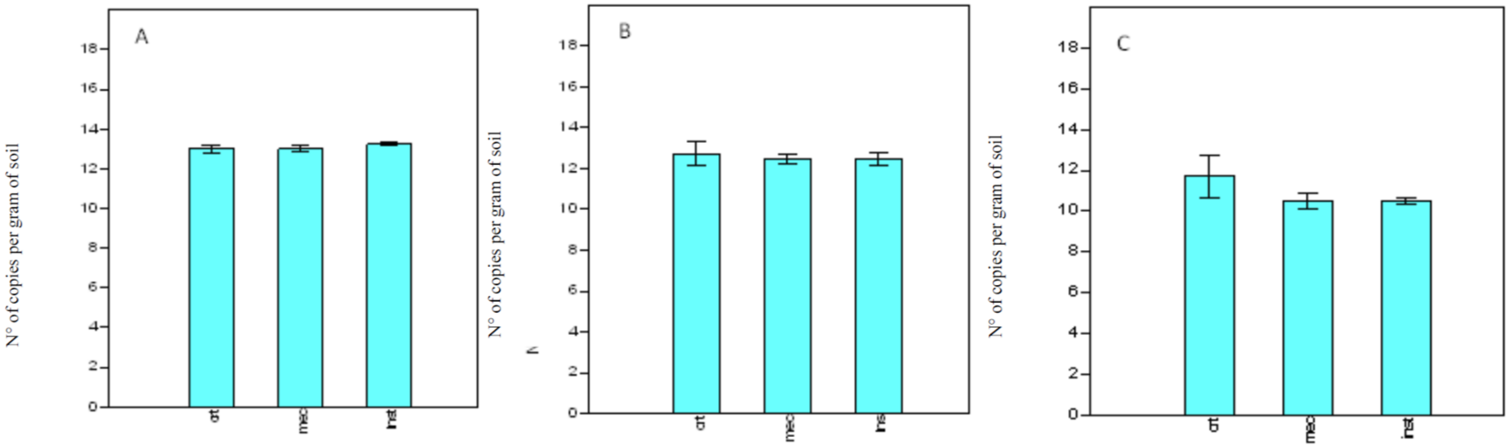

Fig S2. Abundance (n=10) of the16S rRNA gene. (A) Pre-Flowering plant stage; (B) Flowering plant stage; (C) Post Flowering plant stage. The bar on the chart is the mean standard error with 95% interval.

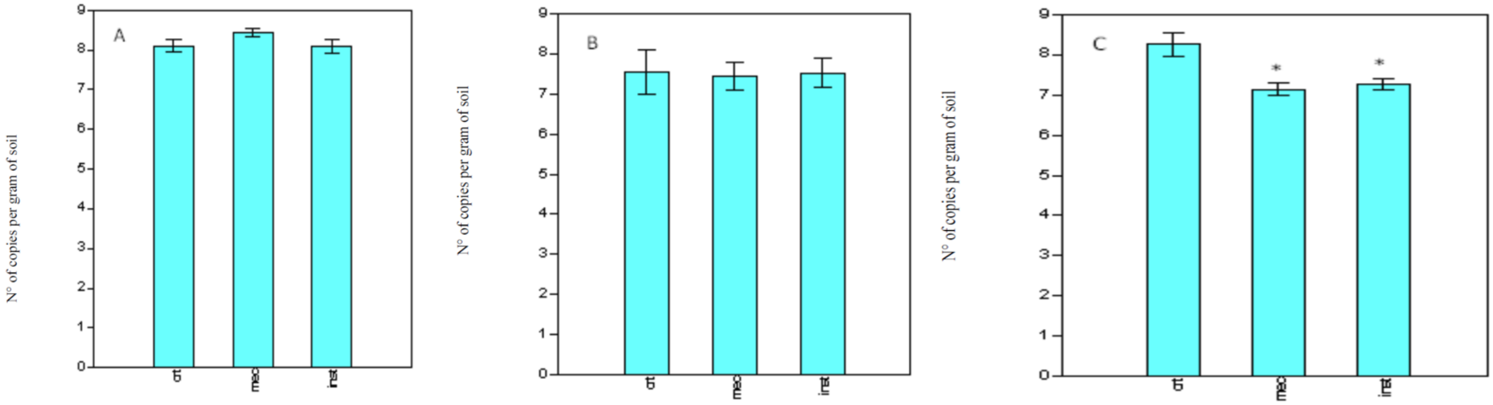

Fig S3. Abundance (n=10) of the Intergenic ITS region among the 18S and 5.8S rRNA gene. (A) Pre-Flowering plant stage; (B) Flowering plant stage; (C) Post Flowering plant stage. The bar on the chart is the mean standard error with 95% interval. (*) significant to $p < 0.05$.
